# Essential role of Na^+^/Ca2^+^ exchanger 1 in smoking-induced growth and migration of esophageal squamous cell carcinoma

**DOI:** 10.18632/oncotarget.11695

**Published:** 2016-08-30

**Authors:** Jiexia Wen, Yan Pang, Tao Zhou, Ximing Qi, Min Zhao, Bin Xuan, Xiangcai Meng, Yunsheng Guo, Qingbin Liu, Huagang Liang, Yang Li, Hui Dong, Yimin Wang

**Affiliations:** ^1^ Department of Central Laboratory, First Hospital of Qinhuangdao, Hebei Medical University, Qinhuangdao, Hebei, China; ^2^ Department of General Surgery, First Hospital of Qinhuangdao, Hebei Medical University, Qinhuangdao, Hebei, China; ^3^ Department of Medicine, University of California, San Diego, California, USA; ^4^ Department of Pathology, First Hospital of Qinhuangdao, Hebei Medical University, Qinhuangdao, Hebei, China; ^5^ Department of Thoracic Surgery, First Hospital of Qinhuangdao, Hebei Medical University, Qinhuangdao, Hebei, China

**Keywords:** Na^+^/Ca2^+^ exchanger 1, calcium signaling, cigarette smoking, esophageal squamous cell carcinoma, tumorigenesis

## Abstract

Tobacco-derived carcinogen 4-(methylnitrosamino)-1-(3-pyridyl)-1-butanone (NNK) is a major environmental risk factor for the pathogenesis of human esophageal squamous cell carcinoma (ESCC). However, the molecular mechanisms by which tobacco induces ESCC are not well understood. Na+/Ca2+ exchanger 1 (NCX1) is a plasma membrane transporter protein that plays an essential role in maintaining cytosolic Ca^2+^ ([Ca^2+^]_cyt_) homeostasis under physiological conditions and is implicated in tumorigenesis as well. In this study, we found that NCX1 expression was significantly higher in ESCC primary tissues compared to the noncancerous tissues and was overexpressed in tumor samples from the smoking patients. The expression of NCX1 proteins was also significantly higher in human ESCC cell lines compared to normal esophageal epithelial cell line. Moreover, NNK potentiated the [Ca^2+^]_cyt_ signaling induced by removal of extracellular Na^+^, which was abolished by KB-R7943 or SN-6. NNK dose-dependently promoted proliferation and migration of human ESCC cells induced by NCX1 activation. Therefore, NCX1 expression correlates with the smoking status of ESCC patients, and NNK activates the Ca^2+^ entry mode of NCX1 in ESCC cells, leading to cell proliferation and migration. Our findings suggest NCX1 protein is a novel potential target for ESCC therapy.

## INTRODUCTION

Esophageal cancer is the sixth leading cause of cancer-related mortality in the world [1–3], incurring more than 456,000 new cases per year worldwide [3], and its prognosis remains poor [2]. Esophageal squamous cell carcinoma (ESCC), accounting for about 80% of esophageal cancer, is notoriously aggressive in nature and spreads by a variety of pathways, including direct extension, lymphatic spread and hematogenous metastasis [4–6]. Since ESCC is characterized by poor survival rates and resistance to radiochemotherapy [7–9], novel therapeutic strategies are required urgently to treat this devastating disease. Although the detailed pathogenesis of ESCC is currently unclear, it has been generally accepted that ESCC results from an interaction between the genetic and environmental factors [1, 10], and the excessive use of tobacco is strongly considered one of the principal factors in the etiology of ESCC.

Cigarette smoking is a serious public health issue and is responsible for millions of deaths worldwide. Cigarette smoking is a major environmental risk factor for mortality of many cancers, including lung cancer, esophageal cancer, liver cancer, gastric cancer and so on [11]. The risk of esophageal cancer has been reported to correlate with the number of cigarettes smoked per day and the duration of smoking. After ingestion of tobacco carcinogens, such as 4-(methylnitrosamino)-1-(3-pyridyl)-1-butanone (NNK), a tobacco-specific nitrosamine, they directly contact with the esophageal epithelium, possibly resulting in tumorigenesis finally. Although cigarette smoking is the most consistent environmental risk factor for ESCC [1, 10], the cellular and molecular mechanisms by which tobacco carcinogens induce ESCC remain poorly understood to date.

Cytosolic free Ca^2+^ ([Ca^2+^]_cyt_) is a ubiquitous cell messenger, and Ca^2+^ entry is a critical component of Ca^2+^ signaling in human physiology. However, dysregulation of Ca^2+^ signaling is involved in tumorigenesis and tumor progression, such as metastasis, invasion and angiogenesis [12, 13]. An alteration of the expression and/or function of Ca^2+^ regulators plays an important role in the process of tumorigenesis [14, 15]. A number of studies found that [Ca^2+^]_cyt_ is elevated in a variety of human cancer cells [16, 17], but a reduction of [Ca^2+^]_cyt_ *via* blockade of Ca^2+^ entry suppress cancer cell growth, suggesting that remodeling of [Ca^2+^]_cyt_ homeostasis might be valuable in cancer therapy [18]. Therefore, it is important to understand the mechanisms by how [Ca^2+^]_cyt_ homeostasis is altered in cancer cells.

Cellular [Ca^2+^]_cyt_ homeostasis is precisely controlled by multiple proteins, including the plasma membrane Na^+^/Ca^2+^ exchanger (NCX). NCX is a family of membrane transporter that operates in either a forward mode (3 Na^+^ entry and 1 Ca^2+^ exit) or a reverse mode (3 Na^+^ exit and 1 Ca^2+^ entry), depending on the electrochemical gradient of Na^+^ and Ca^2+^ and membrane potential [19–21]. NCX1 is expressed in many kinds of mammalian cells [19], including gastrointestinal epithelial cells [22–24]. Since these non-excitable cells may not functionally express voltage-operated Ca^2+^ channels that mainly mediate Ca^2+^ entry in excitable cells, other Ca^2+^ entry pathways, such as NCX1 may fulfill this function [24, 25]. Although NCX1 have been described in gastrointestinal epithelium cells, little is known about its expression and function in human ESCC cells.

Therefore, the aims of the present study were to characterize NCX1 in human ESCC cells and to investigate its role in the pathogenesis of ESCC. We demonstrate for the first time that NCX1 plays an essential role in cigarette component (NNK)-induced proliferation and migration of ESCC cells. Our findings suggest that NCX1 may be a novel potential target for human ESCC therapy.

## RESULTS

### Expression of NCX1 is enhanced in primary ESCC tissues

**Figure 1:**
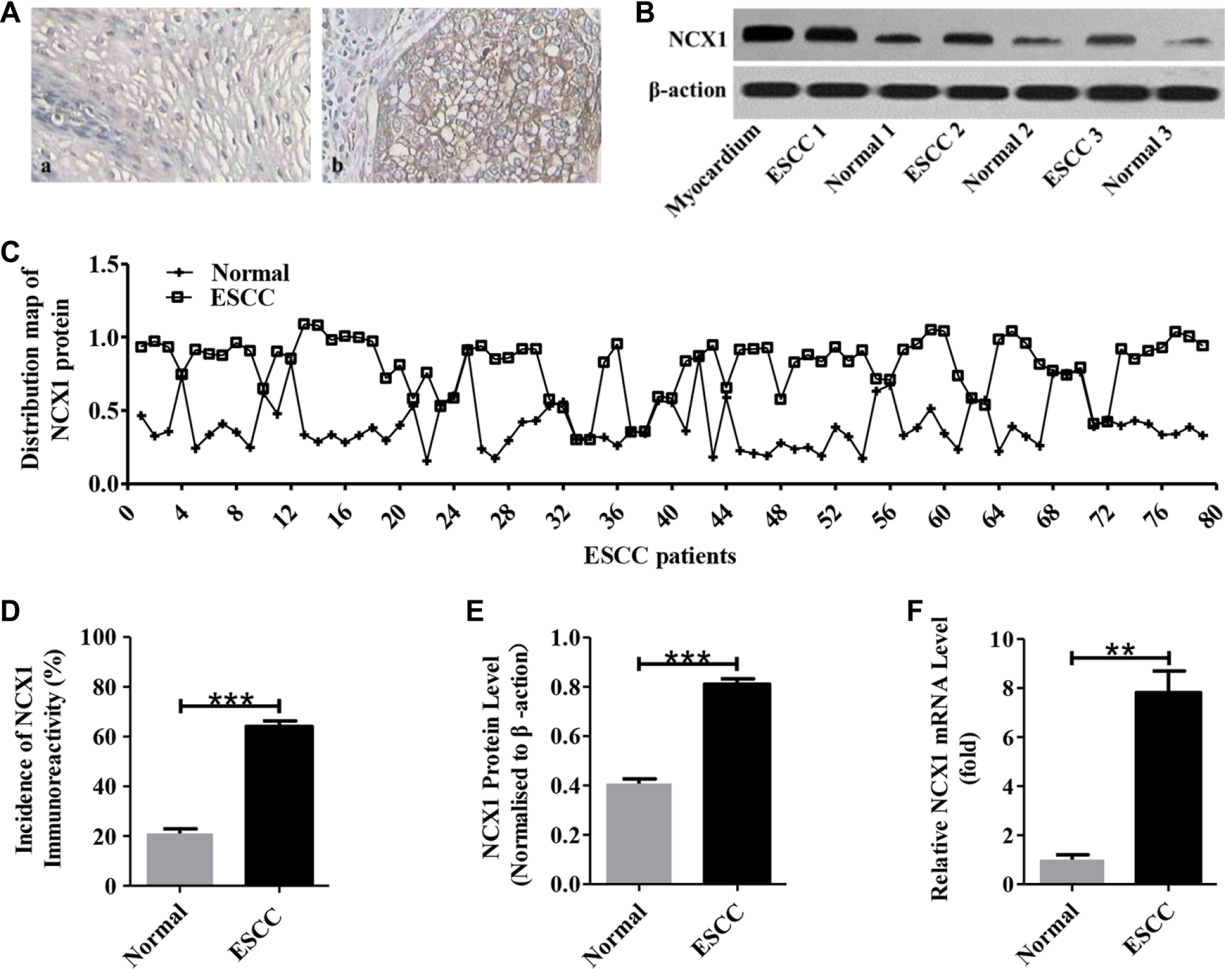
Enhanced expression of NCX1 in primary human ESCC tissues compared with noncancerous normal tissues (**A**) Representative immunohistochemistry analysis for NCX1 proteins in human ESCC tissues (**b**) and their paired noncancerous normal tissues (**a**). Original magnifications: ×400. (**B**) Representative Western blot analysis for NCX1 (top) and β-actin (bottom) in human ESCC tissues and their paired noncancerous normal tissues. Myocardium was used as a positive control, and β-actin was used as an internal standard. (**C**) Distribution map of NCX1 protein in human ESCC tissues and their paired noncancerous normal tissues (*n* = 79). (**D**) A summary of the incidence of NCX1 immunoreactivity in human ESCC tissues and their paired noncancerous normal tissues (*n* = 79). (**E**) A summary of Western blot data comparing the expression of NCX1 proteins in human ESCC tissues and their paired noncancerous normal tissues (*n* = 3). (**F**) A summary of qPCR data comparing the expression of NCX1 at transcriptional level in human ESCC tissues and their paired noncancerous normal tissues (*n* = 3). *P < 0.05, **P < 0.01 or ***P < 0.001 *vs.* normal (paired noncancerous normal tissues).

Expression of NCX1 was demonstrated previously in mammalian gastrointestinal epithelial cells [22–24], however little is known about its expression in human ESCC tissues. Here, we demonstrated that both the transcripts and proteins of NCX1 were overexpressed in human ESCC tissues (Figure 1). After immunohistochemistry analysis of NCX1 proteins on 79 biopsy samples of ESCC and their paired noncancerous tissues, we found that the proportion of NCX1 positive cells was significantly higher in ESCC tissues compared with noncancerous tissues (Figure 1A and 1D). Meanwhile, NCX1 proteins in biopsy tissues were determined by Western blotting and the same trend was found (Figure 1B, 1C and 1E). The mRNA expression of NCX1 in ESCC tissues was also higher compared with noncancerous tissues (Figure 1F). Thus, our data indicate that NCX1 expression at the levels of transcripts and proteins is enhanced in human primary ESCC tissues.

### High expression of NCX1 correlates with the smoking status of ESCC patients

**Figure 2:**
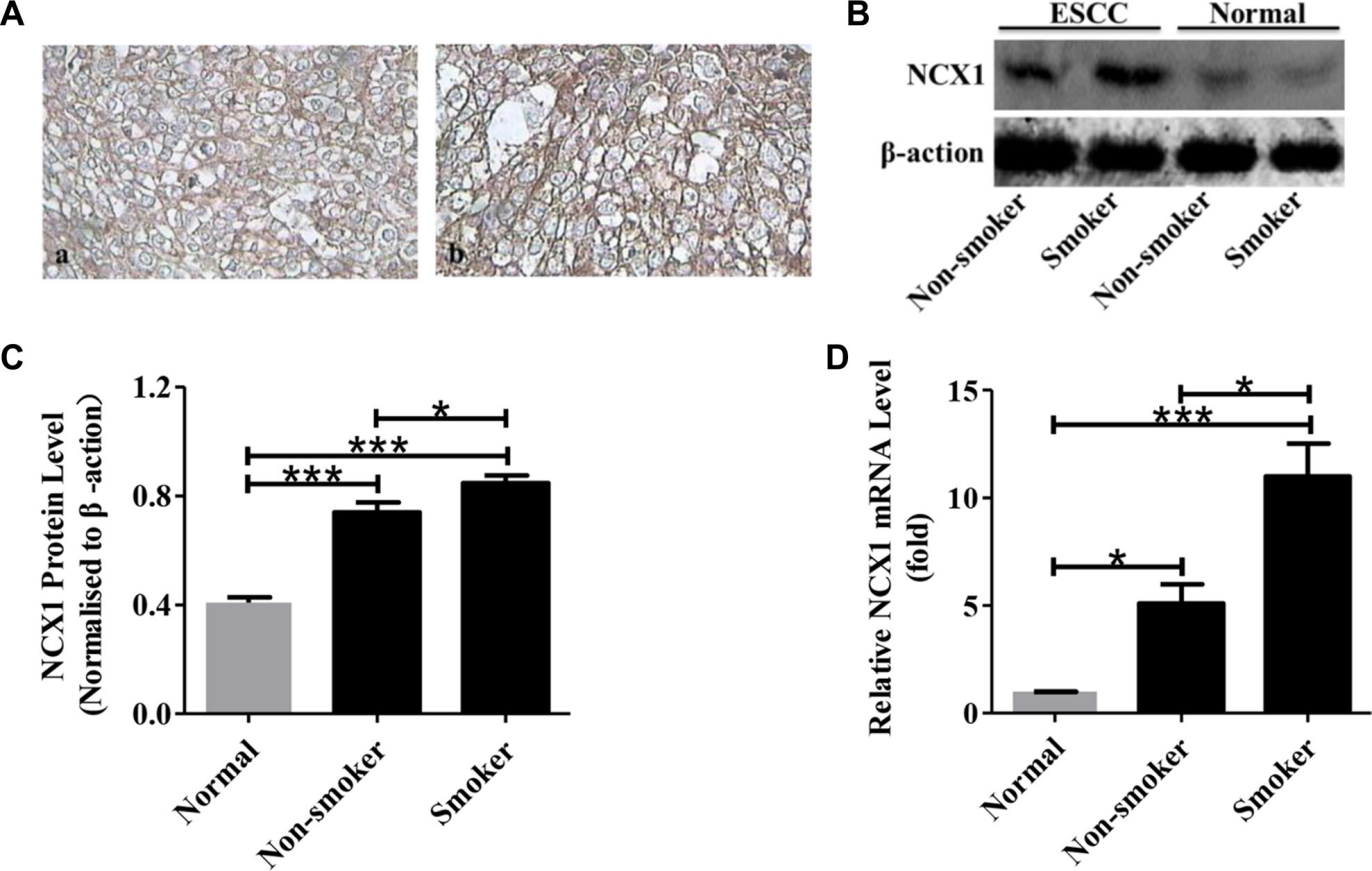
Relationship between NCX1 expression and smoking status of ESCC patients (**A**) Representative immunohistochemistry analysis for NCX1 proteins in human ESCC tissues of non-smokers (**a**) and smokers (**b**). Original magnifications: ×400. (**B**) Representative Western blot analysis for NCX1 (top) and β-actin (bottom) in human ESCC tissues of smokers or non-smokers and their paired noncancerous normal tissues. β-actin was used as an internal standard. (**C**) A summary of Western blot data comparing the expression of NCX1 proteins in human ESCC tissues of smokers and non-smokers (*n* = 3). (**D**) A summary of qPCR data comparing the expression of NCX1 at transcriptional level in human ESCC tissues of smokers and non-smokers (*n* = 3). *P < 0.05, **P < 0.01 or ***P < 0.001 *vs.* normal (paired noncancerous normal tissues).

Since excessive use of tobacco plays a key role in the initiation and promotion of smoking-related malignancy [10], we tested whether a relationship between NCX1 expression and smoking status exists in ESCC patients. As shown in Figure 2A, expression levels of NCX1 protein were higher in ESCC samples from smoking patients compared with those from nonsmoking patients. The chi-square test was also performed to compare the expression levels of NCX1 protein between them. There is a significant difference in the expression of NCX1 protein between the ESCC samples from smoking and nonsmoking patients (Table 1). The expression of NCX1 protein in the ESCC samples from smoking and nonsmoking patients was also determined by Western blotting and the same trend was found (Figure 2B and 2C). Meanwhile, we used qPCR to compare the expression of NCX1 mRNA in the ESCC between smoking and nonsmoking patients and found the expression was higher in ESCC samples from smoking patients (Figure 2D). Consequently, the expression levels of NCX1 correlate with the smoking status of ESCC patients.

**Table 1:** Relationship between NCX1 protein expression and smoking status of ESCC patients

	Non-smoker [Number of cases (%)]	Smoker [Number of cases (%)]	χ^2^	*P* value
NCX1 expression	19	34		
High			4.665	0.031
NCX1 expression	16	10		< 0.05
Low				

### Expression of NCX1 proteins is enhanced in human ESCC cells

**Figure 3:**
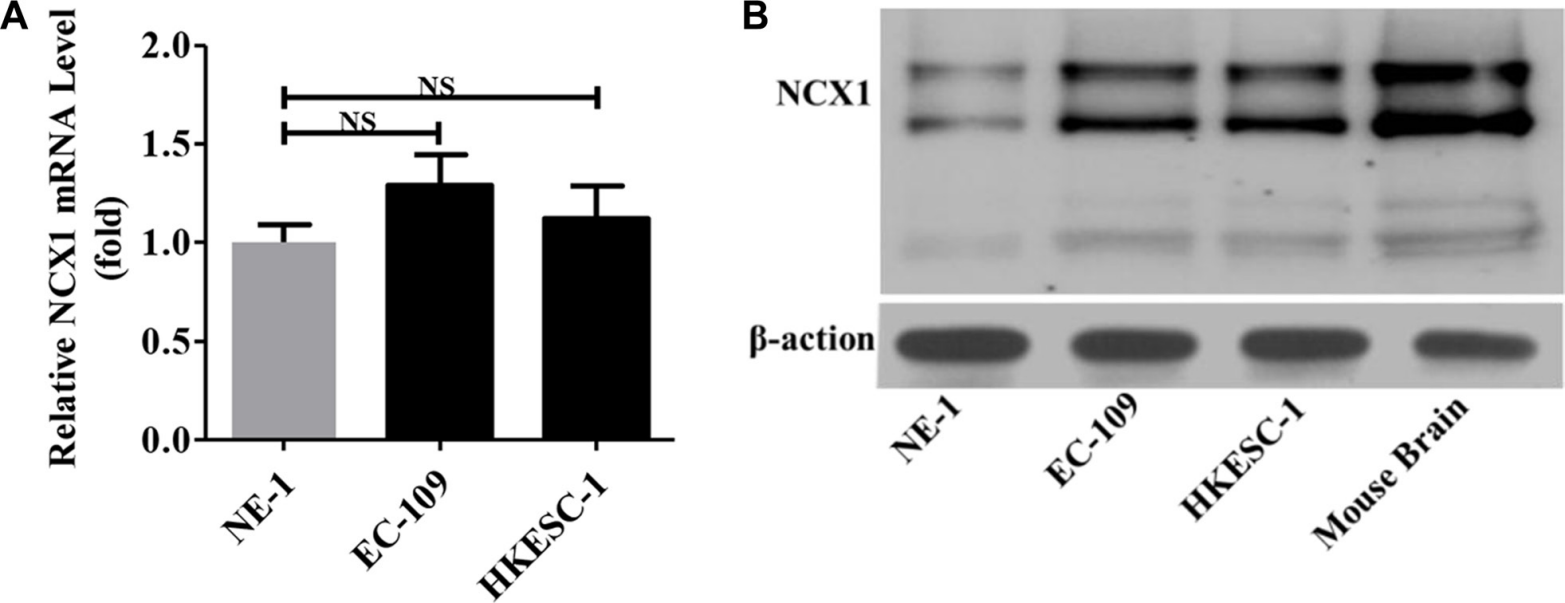
Expression levels of NCX1 in normal human esophageal epithelial cell line (NE-1) and human ESCC cell lines (EC-109 and HKESC-1) (**A**) The qPCR was performed to detect the expression of NCX1 at transcriptional level in cells (*n* = 3). NS: no significant differences. (**B**) Western blot analysis was performed to detect the expression of NCX1 at protein level in cells (*n* = 3). Mouse brain was used as a positive control, and β-actin was used as an internal standard.

We used qPCR to compare the expression levels of NCX1 mRNA between normal human esophageal cell line (NE-1 cells) and human ESCC cell lines (EC-109 and HKESC-1 cells). As shown in Figure 3A, the expression levels of NCX1 mRNA in EC-109 and HKESC-1 cells were slightly higher than that in NE-1 cells. However, when Western blot analysis was performed to compare the expression levels of NCX1 proteins, it was found that NCX1 proteins were significantly higher in EC-109 and HKESC-1 cells compared with NE-1 cells (Figure 3B). Thus, expression of NCX1 proteins is enhanced in human ESCC cells.

### Functional activity of NCX1 is detected in human esophageal cells

**Figure 4:**
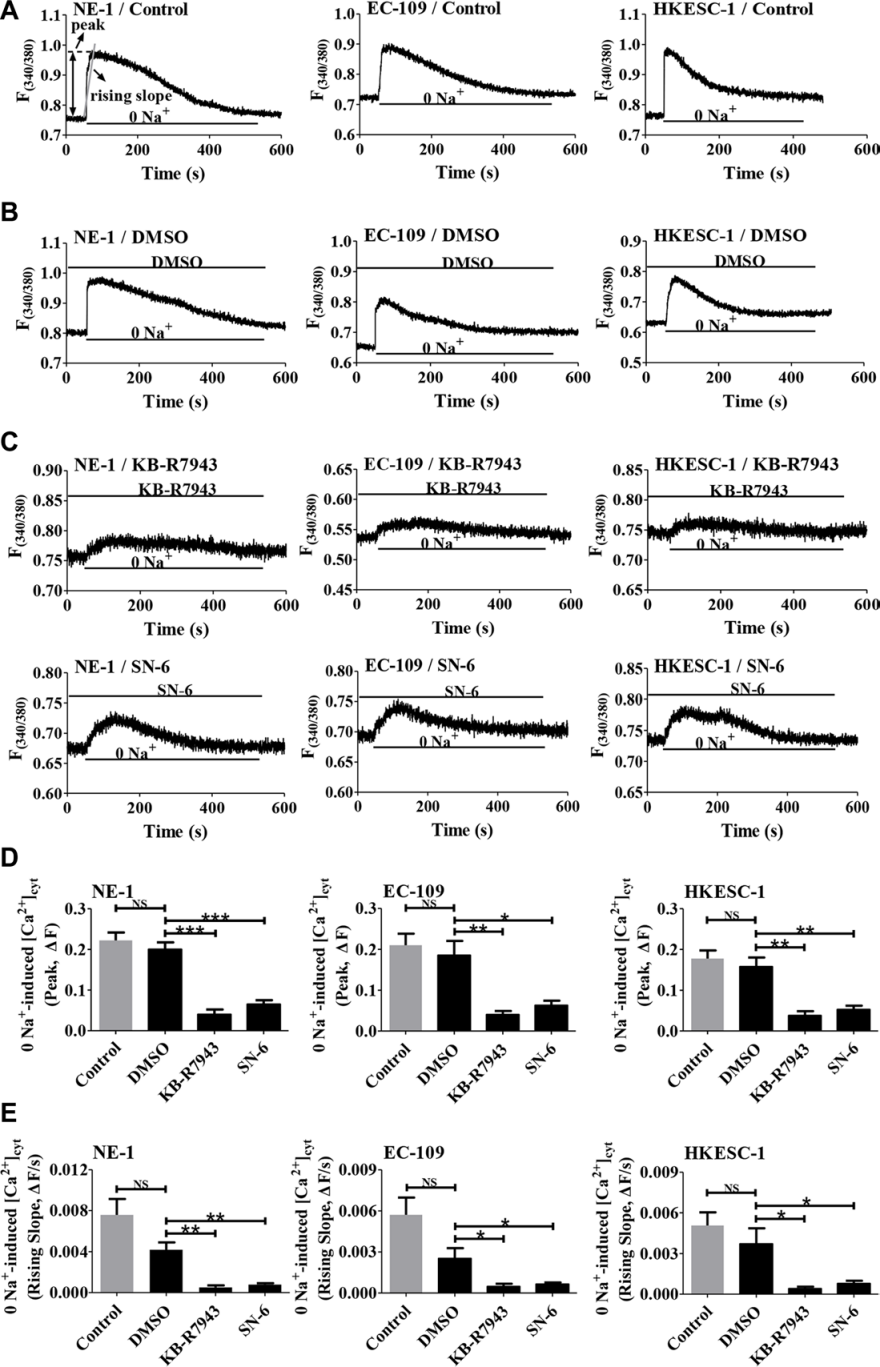
The operation of the Ca^2+^ entry mode of NCX1 in human esophageal cell lines (**A**) Representative original tracings showing basal [Ca^2+^]_cyt_ in normal physiological salt solutions (140 mM Na^+^) and a rapid increase in [Ca^2+^]_cyt_ after removal of Na^+^ (0 Na^+^) in normal human esophageal epithelial cell line (NE-1) and ESCC cell lines (EC-109 and HKESC-1). F_(340/380)_, fluorescence ratio at the excitation of 340/380 nm. Analysis on the peaks and the rising slopes of [Ca^2+^]_cyt_ was also described in A. (**B**) Representative original tracings showing the effect of 0.1% DMSO, the solvent of all drugs, on 0 Na^+^-induced Ca^2+^ entry in esophageal cells. (**C**) Representative original tracings showing the effects of KB-R7943 (10 μM) and SN-6 (10 μM) on 0 Na^+^-induced Ca^2+^ entry in esophageal cells. (**D**) Summary data comparing the effects of DMSO, KB-R7943 and SN-6 on 0 Na^+^-induced peak of [Ca^2+^]_cyt_ in esophageal cells (*n* = 8). (**E**) Summary data comparing the effects of DMSO, KB-R7943 and SN-6 on 0 Na^+^-induced rising slope of [Ca^2+^]_cyt_ in esophageal cells (*n* = 8). NS: no significant differences. *P < 0.05, **P < 0.01 or ***P < 0.001 *vs.* control or 0.1% DMSO.

After expression of NCX1 was identified, we sought to characterize their function in human esophageal cells. To test whether NCX1 functions in the Ca^2+^ entry mode to transport external Ca^2+^ into cell, human esophageal cells (NE-1, EC-109 and HKESC-1) were initially superfused with normal solution, and afterwards switched to the solution without Na^+^ (0 Na^+^ by replacing Na^+^ with equimolar Li^+^ to increase a driving force for operation of the Ca^2+^ entry mode of NCX1). As shown in Figure 4A, a switch to 0 Na^+^ solution caused a rapid increase in [Ca^2+^]_cyt_. We analyzed both peaks and rising slopes of Ca^2+^ entry to quantitatively describe the NCX1 activity in the Ca^2+^ entry mode in cells. Since DMSO (0.1% of final concentration) was used as the solvent of all drugs, its effect was first tested, but it did not affect 0 Na^+^-induced [Ca^2+^]_cyt_ signaling (Figure 4B). However, after extracellular application of KB-R7943 (10 μM) and SN-6 (10 μM), selective inhibitors of NCX1 especially in its Ca^2+^ entry mode, 0 Na^+^-induced [Ca^2+^]_cyt_ was significantly attenuated (Figure 4C). Figure 4D and 4E summarize the inhibitory effects of KB-R7943 and SN-6 on 0 Na^+^-induced peaks and rising slopes of Ca^2+^ entry in the cells. These findings clearly demonstrate that NCX1 functions in the Ca^2+^ entry mode to transport external Ca^2+^ into human esophageal cells.

### NNK enhances higher NCX1 expression in human ESCC cells

**Figure 5:**
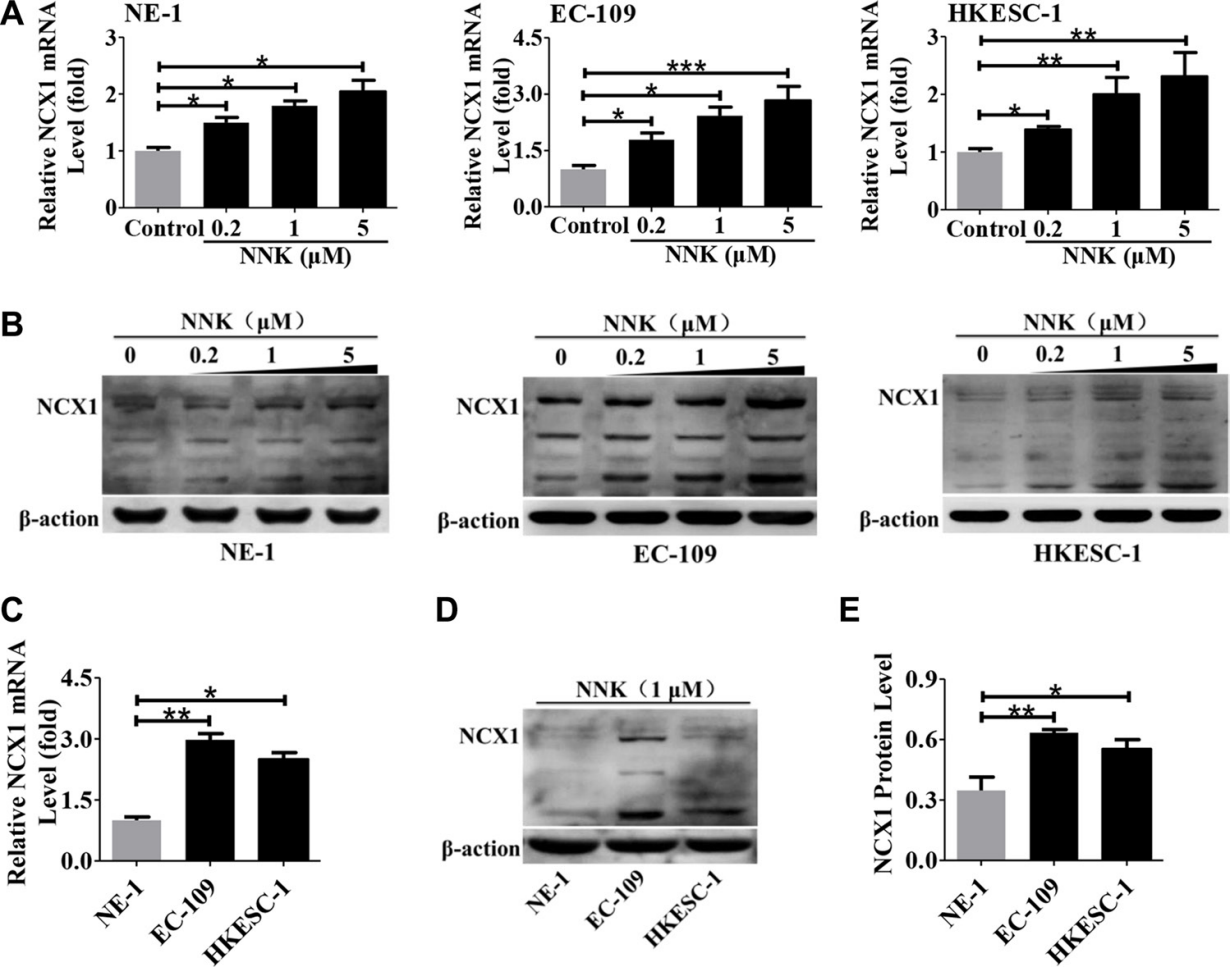
Effect of NNK on NCX1 expression in human esophageal cell lines (**A**) Expression levels of NCX1 transcript in NE-1, EC-109 and HKESC-1 cells were dose-dependently upregulated by NNK treatment for 16 h (*n* = 3). (**B**) Expression level of NCX1 protein in cells was significantly upregulated by NNK treatment in a dose-dependent manner (*n* = 3). (**C**) A summary data comparing NNK-enhanced transcript expression of NCX1 in NE-1, EC-109 and HKESC-1 cells (*n* = 3). (**D**) Representative Western blot analysis of NCX1 protein expression in cells (*n* = 3). (**E**) A summary of Western blot data comparing NNK-enhanced protein expression of NCX1 in NE-1, EC-109 and HKESC-1 cells (*n* = 3). *P < 0.05, **P < 0.01 or ***P < 0.001.

To investigate whether NNK stimulates NCX1 expression, three human esophageal cell lines (NE-1, EC-109 and HKESC-1 cells) were treated with different concentrations of NNK (0.2, 1, and 5 μM) for 16 h, and then the expression levels of NCX1 were analyzed by qPCR and Western blotting. As shown in Figure 5A and 5B, both expression levels of NCX1 transcripts and proteins in three cell lines were significantly upregulated dose-dependently by NNK. Moreover, NNK stimulated higher expression of NCX1 mRNA and proteins in EC-109 and HKESC-1 cells than in NE-1 cells (Figure 5C, 5D and 5E). These results suggest that NNK enhances higher NCX1 expression in human ESCC cells than in normal esophageal cells.

### NNK enhances higher NCX1 activity in human ESCC cells

**Figure 6:**
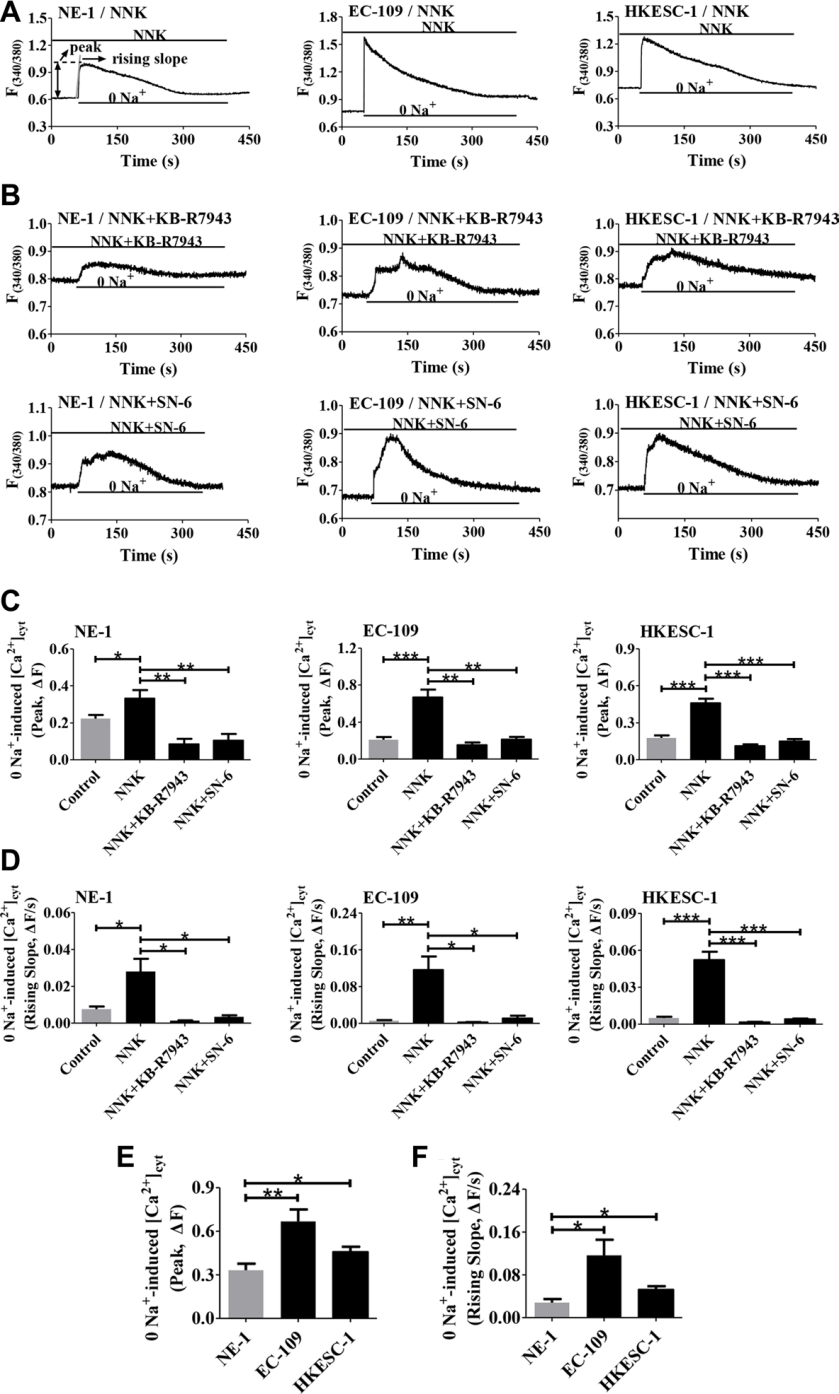
Effect of NNK on NCX1 function in human esophageal cell lines (**A**) Representative original tracings showing removal of Na^+^ (0 Na^+^)-induced a rapid increase in [Ca^2+^]_cyt_ in NNK-pretreated NE-1, EC-109 and HKESC-1 cells. (**B**) Representative original tracings showing the effects of KB-R7943 (10 μM) and SN-6 (10 μM) on 0 Na^+^-induced increase in [Ca^2+^]_cyt_ in NNK-pretreated cells. (**C** and **D**) Summary data showing the effects of KB-R7943 and SN-6 on 0 Na^+^-induced peaks and rising slopes of [Ca^2+^]_cyt_ in NNK-treated cells (*n* = 8). (**E** and **F**) Summary data comparing the effect of NNK on 0 Na^+^-induced peaks and rising slopes of [Ca^2+^]_cyt_ in NE-1, EC-109 and HKESC-1 cells (*n* = 8). For all NNK-pretreated cells, they were pretreated with NNK (1 μM) for 16 h. *P < 0.05, **P < 0.01 or ***P < 0.001.

We subsequently characterized NCX1 function in human esophageal cells. As shown in Figure 6A, pretreatment of cells with NNK (1 μM) for 16 h enhanced the activity of the Ca^2+^ entry mode of NCX1 in human esophageal cells. Moreover, KB-R7943 and SN-6 significantly prevented NNK-induced Ca^2+^ influx in these cells (Figure 6B). Figure 6C and 6D summarize the NNK-induced peaks and rising slopes of Ca^2+^ entry in the cells and the inhibitory effects of KB-R7943 and SN-6 on them. Interestingly, NNK induced greater NCX1 activity in human ESCC cells (EC-109 and HKESC-1) than in normal esophageal cells (NE-1) (Figure 6E and 6F). Therefore, NNK enhances not only higher NCX1 expression in human ESCC cells but NCX1 activity as well.

### NCX1 is involved in NNK-promoted proliferation of human esophageal cells

**Figure 7:**
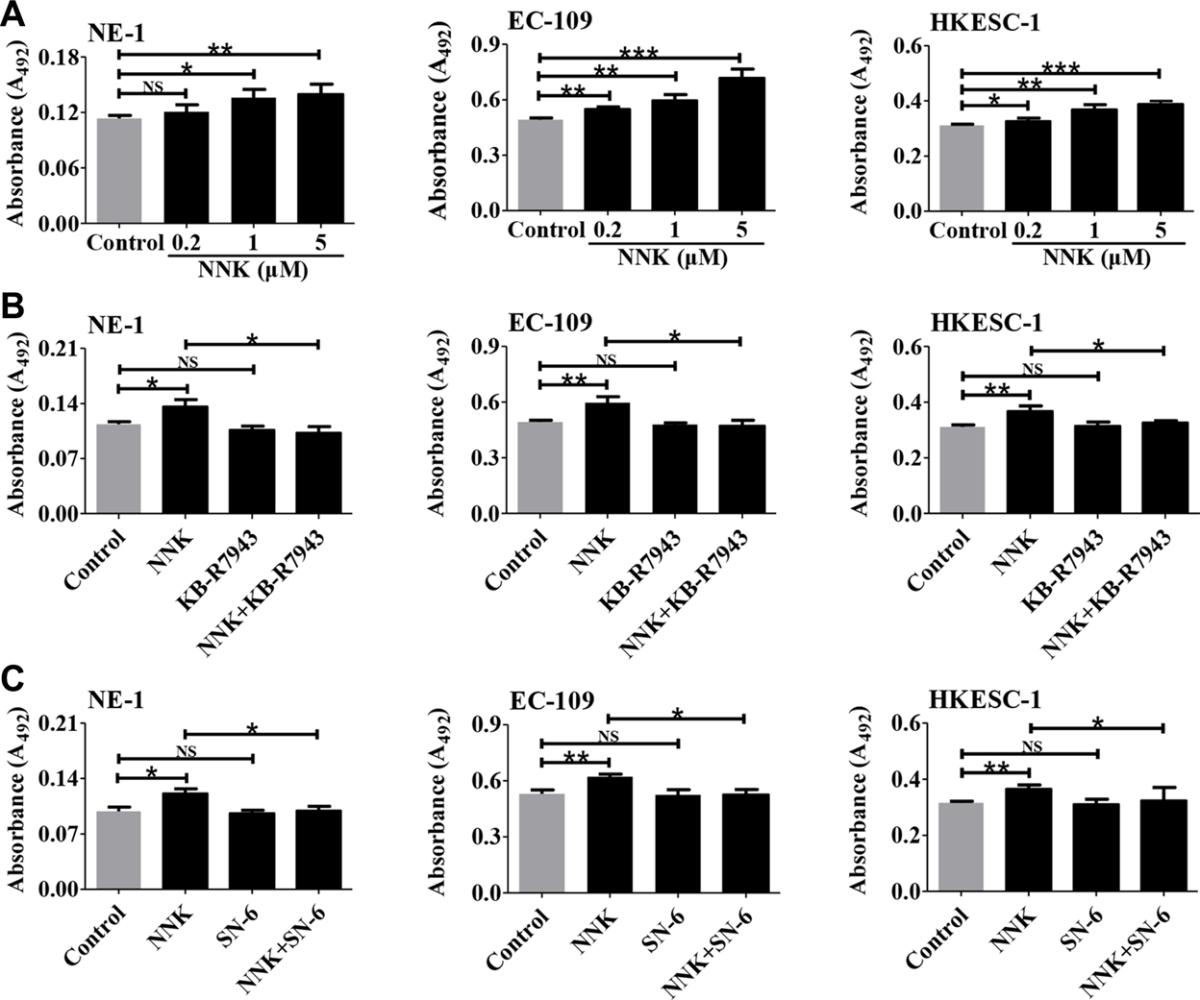
The involvement of NCX1 in NNK-promoted proliferation of human esophageal cell lines (**A**) Summary data showing the effect of NNK at different concentrations on proliferation of NE-1, EC-109, and HKESC-1 cells (*n* = 4). (**B**) Summary data showing the effect of KB-R7943 (10 μM) on NNK (1 μM)-promoted cell proliferation (*n* = 4). (**C**) Summary data showing the effect of SN-6 (10 μM) on NNK (1 μM)-promoted cell proliferation (*n* = 4). NS: no significant differences. *P < 0.05, **P < 0.01 or ***P < 0.001.

Since NCX1 plays an essential role in controlling Ca^2+^ signaling that is implicated in tumorigenesis [21], we tested the effect of NNK on proliferation of human esophageal cells and then the role of NCX1 in NNK-mediated cell proliferation. NNK significantly promoted proliferation of esophageal cells in a concentration-dependent manner (Figure 7A). However, NNK-promoted proliferation of human esophageal cells was abolished by KB-R7943 and SN-6 (Figure 7B and 7C). Therefore, NNK promotes proliferation of human esophageal cells through NCX1 activation.

### NCX1 is involved in NNK-promoted migration of human ESCC cells

**Figure 8:**
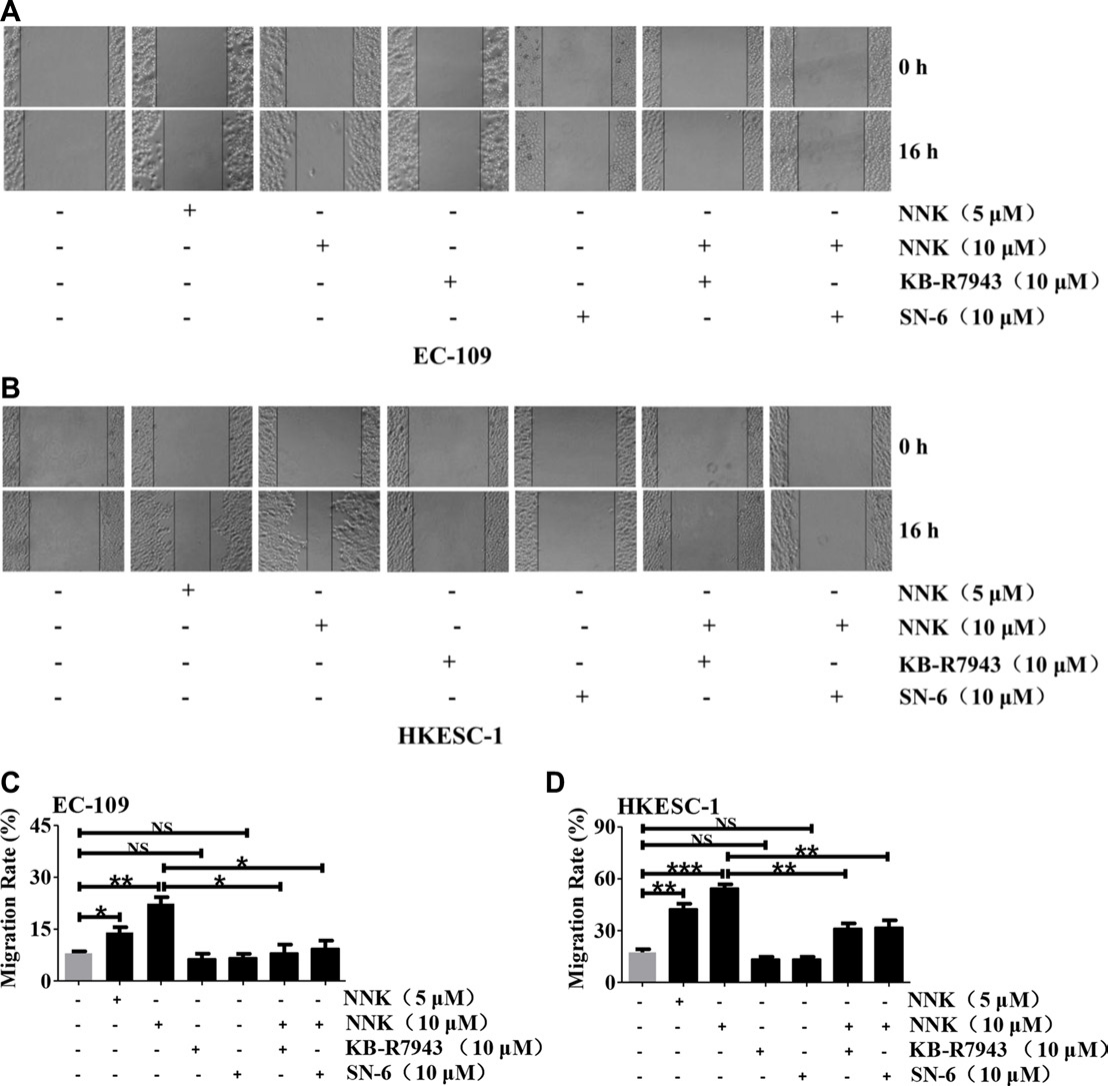
The involvement of NCX1 in NNK-promoted migration of human ESCC cells Representative scratch assays showing the effect of NNK at different concentrations on cell migration and the effects of KB-R7943 and SN-6 on NNK-promoted migration of EC-109 cells (**A**) and HKESC-1 cells (**B**). Summary data showing the effect of NNK at different concentrations on cell migration and the effects of KB-R7943 and SN-6 on NNK (10 μM) -promoted migration of EC-109 cells (**C**) and HKESC-1 cells (**D**). *N* = 3, NS: no significant differences, *P < 0.05, **P < 0.01 or ***P < 0.001.

We used scratch assay to test whether NNK regulates migration of human ESCC cells through NCX1 activation. EC-109 and HKESC-1 cells were pre-treated with mitomycin C (30 μM) to prevent cell proliferation. As shown in Figure 8A and 8B, after the cells were pretreated with NNK (10 μM), migration was increased by 22% for EC-109 (Figure 8C) and 54% for HKESC-1 cells (Figure 8D), respectively. Although KB-R7943 or SN-6 alone did not alter cell migration, they significantly reversed NNK-induced migration of EC-109 and HKESC-1 cells (Figure 8C and 8D). Thus, NNK promotes migration of human ESCC cells *via* NCX1 activation.

## DISCUSSION

ESCC is the most prevalent histological type of esophageal cancer worldwide, and its 5-year survival rate is about 12% to 15% only [26, 27]. Unfortunately, the pathogenic mechanisms of ESCC remain poorly understood although ESCC is found to be environment-dependent. Specifically, tobacco smoking is the environmental risk factor for ESCC, and it may cause genetic and epigenetic changes to eventually lead to ESCC [1, 10]. However, the detailed molecular mechanisms by how tobacco smoking induces the devolvement of ESCC have not been fully understood so far.

In the present study, we demonstrate for the first time that NCX1 is involved in the development of ESCC because: 1) NCX1 expression was significantly higher in human primary tissues of ESCC and cancer cells compared to esophageal noncancerous tissues and normal cells; 2) NCX1 expression was positively associated with tobacco consumption of ESCC patients; 3) NCX1 expression in esophageal normal and cancer cells was dose-dependently enhanced by NNK treatment; 4) NNK stimulated higher expression of NCX1 mRNA and proteins in ESCC cells than in normal cells; and 5) NNK promoted proliferation and migration of ESCC cells through NCX1-mediated Ca^2+^ entry.

NCX1 plays an important role in controlling [Ca^2+^]_cyt_ homeostasis in various types of human cells. It can exchange Na^+^ and Ca^2+^ in either direction depending on transmembrane electrochemical gradients [19, 20]. Although the primary physiological function of NCX1 is to expel Ca^2+^ from the cell *via* Ca^2+^ exit mode, NCX1 can operate in Ca^2+^ entry mode to raise [Ca^2+^]_cyt_ under some pathological status [21, 28]. Disruption of [Ca^2+^]_cyt_ homeostasis induced by abnormal [Ca^2+^]_cyt_ regulators, especially enhanced Ca^2+^ entry mode of NCX1, has been detected in some cancers, such as pancreatic cancer [18], breast cancer [29], penile cancer [30], and cervical cancer [31], indicating an significant role of NCX1 in tumorigenesis. However, the role of NCX1-mediated Ca^2+^ signaling in the development of ESCC remains currently unexplored.

It has been reported that under pathological status such as during tumorigenesis, excessive cell metabolism could reduce intracellular pH to activate Na^+^/H^+^ exchanger (NHE), which raises [Na^+^]_cyt_ under the restricted plasma membrane space and induce membrane depolarization [32, 33]. A small increase in [Na^+^]_cyt_ and depolarization can switch on NCX1 to allow sustained Ca^2+^ entry [34, 35]. Indeed, we revealed that NCX1 could function in the Ca^2+^ entry mode to transport external Ca^2+^ into esophageal cells, which was significantly attenuated by selective inhibitors. These results strongly suggest that NCX1 plays an important role in the regulation of [Ca^2+^]_cyt_ homeostasis in ESCC cells. Moreover, we found that NNK dose-dependently stimulated not only NCX1 expression in ESCC but its function as well. These findings indicate that tobacco smoking could cause Ca^2+^ entry through enhanced expression and function of NCX1 to finally result in the pathogenesis of ESCC.

It has been well documented that [Ca^2+^]_cyt_ is a pivotal messenger that regulates many biological functions including cell proliferation and migration [14, 36]. Many ESCC patients die as a result of cancer cell excessive proliferation and metastasis [4–6]. Our study showed that NNK dose-dependently stimulated the proliferation and migration of ESCC cells, which was reversed by NCX1 inhibitors at the concentrations that selectively inhibit NCX1 activity. These findings strongly suggest that NNK promotes proliferation and migration of human ESCC cells likely through NCX1 activation.

**Figure 9:**
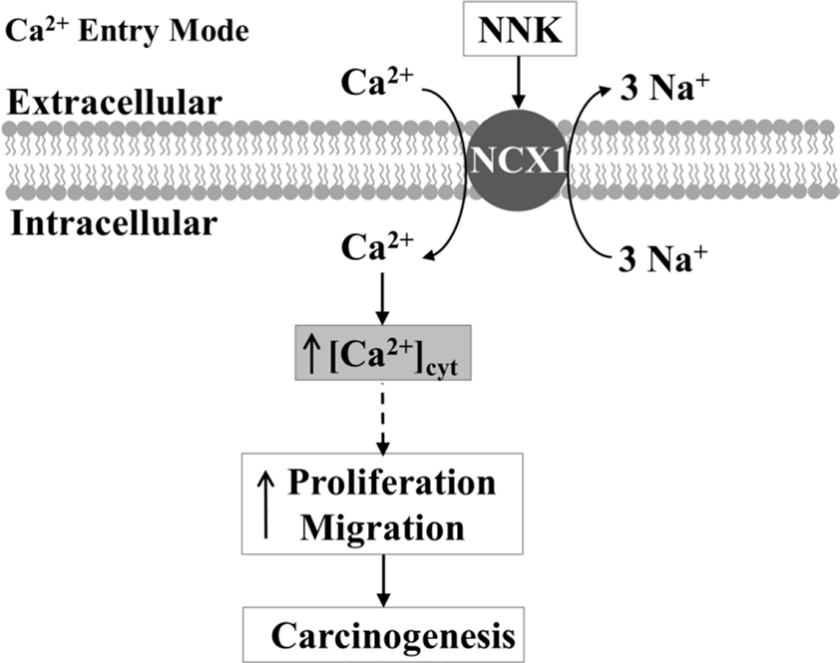
Schematic diagram depicting the proposed mechanisms of esophageal carcinogenesis mediated by [Ca^2+^]_cyt_ rise through the Ca^2+^ entry mode of NCX1 NNK enhances expression and function of NCX1, inducing the sustained inward transportation of Ca^2+^ through the Ca^2+^ entry mode of NCX1. An increase in [Ca^2+^]_cyt_ through NCX1 serves as a major stimulus for proliferation and migration of human ESCC cells.

In conclusion, we demonstrate that NCX1 overexpression is correlated with the smoking status of ESCC patients. NNK may induce an increase in [Ca^2+^]_cyt_ through enhanced expression and function of NCX1 in ESCC cells, finally leading to excessive proliferation and migration. A proposed schematic of NNK-evoked ESCC through the Ca^2+^ entry mode of NCX1 is shown in Figure 9. Although further studies are needed to elucidate the detailed mechanisms by how NCX1-mediated Ca^2+^ entry induces human ESCC, our study has provided a novel perspective that targeting NCX1 may be a potential therapeutic strategy for ESCC.

## MATERIALS AND METHODS

### Materials and reagents

4-(Methylnitrosamino)-1-(3-pyridyl)-1-butanone (NNK) was purchased from Fluka (Buchs, Switzerland). SN-6 and KB-R7943 mesylate were from Tocris Bioscience (Ellisville, MO, USA). Fura-2/AM was from Molecular Probes (Eugene, OR, USA). A solution of NCX1 inhibitor in DMSO (Sigma, St. Louis, MO, USA) and a solution of fura-2/AM in pluronic F-127 (Molecular Probes, Eugene, OR, USA) were prepared. GoScript™ Reverse Transcription System and GoTaq® qPCR Master Mix were purchased from promega (Madison, WI, USA). Mitomycin C was from Roche (Nutley, NJ, USA). All other reagents were purchased from Sigma (St. Louis, MO, USA).

### Clinical samples

Seventy-nine pairs of human ESCC tissues and their paired noncancerous esophageal tissues were obtained from the Department of Pathology, First Hospital of Qinhuangdao of China and The Fourth Hospital of Hebei Province of China. The study was approved by the Ethical Review Boards of the hospitals. ESCC tissues and corresponding noncancerous esophageal tissues were isolated and placed into sterile freezing vials and stored immediately in liquid nitrogen for further use. All samples were histologically reviewed by two pathologists to confirm the diagnosis of ESCC. All patients were classified into smoking group and non-smoking group. Tobacco smoking was defined as smoking at least one cigarette per day for over 1 year [11, 37].

### Cell lines and cell culture

Human normal esophageal epithelial cell line (NE-1 cells) and human ESCC cell lines (EC-109 and HKESC-1 cells) were obtained from American AddexBio Technologies. NE-1 cells were maintained in Karatinocyte-SFM (Gibco/BRL, Carlsbad, CA, USA). EC-109 and HKESC-1 cells were maintained respectively in RPMI1640 (Gibco/BRL, Gaithersburg, MD, USA) and DMEM (Gibco/BRL, Gaithersburg, MD, USA) supplemented with 10% fetal calf serum (FBS, Gibco/BRL, Gaithersburg, MD, USA). These cell lines were cultured in an incubator under a humidified atmosphere of 5% CO_2_−95% air at 37°C and were grown to 70–80% confluence in medium. During the experiments, cells were washed twice in PBS, incubated in serum-free medium, and treated with 0.2, 1, 5, 10 μM NNK [38–42], 0.1% DMSO or medium alone throughout the experiment.

### Immunohistochemistry (IHC)

Five micrometer thick sections of paraffin-embedded tissues were deparaffinized and dehydrated. Endogenous peroxidase activity was blocked by incubating sections in 3% H_2_O_2_. Antigen retrieval was conducted by heating in a pressure cooker filled with 10 mM citrate buffer (pH 6.0) for 10 min. After treatment with 10% normal goat serum for 20 min to block any non-specific reaction, sections were incubated with a rabbit anti-human antibody against NCX1 (dilution 1:500, Swant) over-night at 4°C. Sections were then incubated with a biotinylated secondary antibody (anti-rabbit immunoglobulin) at room temperature for 30 min and then with an avidin-biotin-peroxidase complex at room temperature for 30 min. 3, 3′-diaminobenzidine-hydrogen (DAB) was used as chromogen, followed by light hematoxylin counterstaining. Slides omitting primary antibody were used as negative controls. For each sample, at least 3 high power fields and 500 cells were randomly counted. If the lesion was small, with less than 500 cells, all of the cells were counted. The incidence of NCX1 immunoreactivity rate in each sample was expressed as a percentage of all the cells counted. It was considered to be high if the level of NCX1 protein expression rate in the ESCC tissue was above the percentage of corresponding normal esophageal tissue, and low if below the percentage.

### Total RNA extraction and quantitative real-time PCR (qPCR)

Total RNA was extracted from tissues or cell lines using the TRIZAL reagent (Invitrogen, Carlsbad, CA, USA). Lysates of tissues or cells were combined with chloroform and mixed, and the pellets were precipitated with isopropanol and 75% ethanol and then air dried. RNA concentration was quantified on a spectrophotometer (ScanDrop 250, Analytik Jena AG, Jena, Germany) reading dual wave lengths of 260 and 280 nm. RNA was reverse transcribed into complementary DNA (cDNA) according to the manufacturer's instruction (GoScript™ Reverse Transcription System, Promega Corporation). The cDNA was amplified by using GoTaq^®^ qPCR Master Mix (Promega Corporation), gene-specific primers and ABI PRISM 7900HT (Applied Biosystems, Foster City, CA, USA). Quantitative real-time PCR (qPCR) primers of the NCX1 genes were used based on the literature [43]. Primer sequences are listed in Table 2. The expression level of NCX1 was defined from the threshold cycle (Ct), and relative expression level was calculated using the 2^−ΔΔCt^ method after normalization with reference to the expression of β-actin. Each reaction was performed in triplicate.

**Table 2:** Primer sequences for quantitative real-time PCR (qPCR) analysis

Target Gene	Accession No.	Primer Sequence
*NCX1*	NM_021097	Sense 5′-TGTGCATCTCAGCAATGTCA-3′
		Antisense 5′-TTCCTCGAGCTCCAGATGTT-3′
β-actin	BC002409.2	Sense 5′-TGGCACCCAGCACAATGAA-3′
		Antisense 5′-CTAAGTCATAGTCCGCCTAGAAGCA-3′

### Western blotting

The treated cells were washed two times with ice-cold PBS. Cells or tissues then lysed with total lysis buffer containing protease inhibitors (100 μg/ml PMSF). The lysates were then incubated for 15 min with constant shaking at 4°C. The lysates were then scraped into micro-centrifuge tubes, and the samples were centrifuged at 12,000 r/min for 15 min to remove insoluble material. The protein content in each sample was determined and adjusted. Protein samples were mixed with 4× gel loading buffer and boiled for 5 min. Protein samples were separated by 8% SDS-polyacrylamide gel-electrophoresis and transferred to polyvinylidene difluoride (PVDF) membrane (Millipore, Billerica, MA, USA). Membranes were then blocked with a 5% solution of skim milk for 1 hour at 37°C. Then the membranes were washed with TBS for 3 times, followed by further incubation with specific antibodies [NCX1, 1:1000 (Swant) or β-Actin, 1:5,000 (ImmunoWay)]. After being washed with TBS with 1% Tween (TBST) for 3 times, the secondary antibody (Peroxidase-Conjugated AffiniPure Goat Anti-Rabbit IgG, Santa Cruz Biotechnology) was applied to the membrane. After being washed with PBST, the membrane was treated with a chemiluminescent solution (Millipore Corporation) according to manufacturer's instructions. Immunoreactive bands were visualized by ECL Western blotting analysis system (Amersham imager 600, Amersham Biosciences, Piscataway, NJ, USA). Densitometric analysis of the blots was conducted using ImageJ software and β-actin protein levels were used as a control for equal protein loading.

### Measurement of [Ca^2+^]_cyt_ by digital Ca^2+^ imaging

[Ca^2+^]_cyt_ was measured by fura-2/AM fluorescence ratio digital imaging. Briefly, cells grown on coverslips were loaded with 5 μM fura-2/AM dissolved in 0.02% Pluronic F-127 plus 0.1% DMSO in physiological salt solution (PSS, described below) in the dark at room temperature (22°C) for 1 hour and then washed in PSS for 30 min. Thereafter, the coverslips with cells were mounted in a perfusion chamber on a Nikon microscope stage. The ratio of fura-2/AM fluorescence with excitation at 340 or 380 nm (F_340/380_) was followed over time and used the PTI RatioMaster fluorescence system (Photon Technology International, Birmingham, N.J., USA). The corresponding ratios F_(340/380)_ were used to obtain relative intracellular Ca^2+^ concentrations. As a measure for the increase of cytosolic Ca^2+^ activity, the rising slope and peak of the changes in the F_(340/380)_ were determined for each experiment. Average fluorescence values of 5–10 attached cells were recorded. These cells were mostly grown in separate regions with no apparent connection. The experiments were performed more than 8 times. The PSS solution used in digital Ca^2+^ measurement contained the following (in mmol/L): 140 Na^+^, 5 K^+^, 2 Ca^2+^, 149 Cl^−^, 10 HEPES, and 10 glucose. In Na^+^-free PSS, NaCl was replaced by LiCl.

### Cell proliferation assay

For cell proliferation measurement, cells were either pretreated with KB-R7943 (10 μM) or SN-6 (10 μM) for 4 h or no drug treatment, 5 × 10^3^ cells were reseeded into 96-well culture plates, then treated with NNK at the concentrations of 0.2, 1, 5 μM for 24 hours. After incubation, the medium was removed and 20 μl MTT (5 mg/mL, Sigma) was added to each well for 4 hours. The formazan precipitate was dissolved in 200 μl of DMSO and the optical density of each well was read on the plate reader at 492 nm. At least 4 independentents were performed, and the averages were calculated.

### Scratch Assay

Cells were propagated with complete media in 6-well plates, respectively, and allowed to grow to 90% confluence over-night. For scratch assay, cells were pre-treated on the following day with mitomycin C at 10 μg/mL (30 μM) and/or KB-R7943 (10 μM) or SN-6 (10 μM) for 4 h. By mechanically scratching the cell substrate with a pipette tip, characteristically sized wounds were performed in all wells. Remaining adherent cells were washed with PBS, respectively replenished with 1% serum medium, and allowed to stabilize for 1 hour. The cells were treated with 0.1% DMSO with 30 μM mitomycin C for vehicle control, 5 μM NNK with 30 μM mitomycin C, 10 μM NNK with 30 μM mitomycin C, 10 μM KB-R7943 with 30 μM mitomycin C, 10 μM NNK and 10 μM KB-R7943 with 30 μM mitomycin C for 16 hours. Gap closures of marked areas were recorded at 50 magnification using a Leica DMI3000B microscope (Leica Microsystems, Wetzlar, Germany) and Leica Application suite V4 capture software (Leica Microsystems, Wetzlar, Germany). Gap closures were measured over a time course to calculate the migration rate according to the following formula: migration rate = ((gap length at 0 h) − (gap length at 16 h) / (gap length at 0 h)) × 100%. The experiments were performed more than 3 times.

### Statistical analysis

SPSS 13.0 software (Chicago, IL, USA) was used for statistical analysis. The chi-square test was used to determine relationship exists between NCX1 expression and smoking status in ESCC patients. Data were analyzed by Student's *t*-tests for unpaired samples with GraphPad Prism 3.0 (San Diego, CA, USA). The results were presented as mean ± SD unless otherwise stated. A difference with a *P* value of less than 0.05 was considered statistically significant.
